# Spatial Frequency Tuning during the Conscious and Non-Conscious Perception of Emotional Facial Expressions – An Intracranial ERP Study

**DOI:** 10.3389/fpsyg.2012.00237

**Published:** 2012-07-19

**Authors:** Verena Willenbockel, Franco Lepore, Dang Khoa Nguyen, Alain Bouthillier, Frédéric Gosselin

**Affiliations:** ^1^Centre de Recherche en Neuropsychologie et Cognition, Département de Psychologie, Université de MontréalMontréal, QC, Canada; ^2^Centre Hospitalier de I’Université de Montréal, Hôpital Notre-DameMontréal, QC, Canada

**Keywords:** consciousness, emotional facial expressions, spatial frequency

## Abstract

Previous studies have shown that complex visual stimuli, such as emotional facial expressions, can influence brain activity independently of the observers’ awareness. Little is known yet, however, about the “informational correlates” of consciousness – i.e., which low-level information correlates with brain activation during conscious vs. non-conscious perception. Here, we investigated this question in the spatial frequency (SF) domain. We examined which SFs in disgusted and fearful faces modulate activation in the insula and amygdala over time and as a function of awareness, using a combination of intracranial event-related potentials (ERPs), SF Bubbles (Willenbockel et al., 2010a), and Continuous Flash Suppression (CFS; Tsuchiya and Koch, 2005). Patients implanted with electrodes for epilepsy monitoring viewed face photographs (13° × 7°) that were randomly SF filtered on a trial-by-trial basis. In the conscious condition, the faces were visible; in the non-conscious condition, they were rendered invisible using CFS. The data were analyzed by performing multiple linear regressions on the SF filters from each trial and the transformed ERP amplitudes across time. The resulting classification images suggest that many SFs are involved in the conscious and non-conscious perception of emotional expressions, with SFs between 6 and 10 cycles per face width being particularly important early on. The results also revealed qualitative differences between the awareness conditions for both regions. Non-conscious processing relied on low SFs more and was faster than conscious processing. Overall, our findings are consistent with the idea that different pathways are employed for the processing of emotional stimuli under different degrees of awareness. The present study represents a first step to mapping how SF information “flows” through the emotion-processing network with a high temporal resolution and to shedding light on the informational correlates of consciousness in general.

## Introduction

The look on someone’s face can speak volumes. Emotional facial expressions convey a wealth of information, such as cues about a person’s state of mind or warning signs of potentially threatening situations (e.g., reflected by fear) or materials (e.g., reflected by disgust). Human faces and brains are thought to have co-evolved to be efficient transmitters and decoders of emotional signals, respectively (Smith et al., 2005; Schyns et al., 2007, 2009). Moreover, it has been claimed that emotional information from a face can be extracted without the observer’s awareness (see Tamietto and De Gelder, 2010, for a review). Numerous studies have shown that face stimuli rendered “invisible” using techniques such as backward masking (e.g., Smith, in press), binocular rivalry (e.g., Williams et al., 2004), or Continuous Flash Suppression (CFS; e.g., Tsuchiya and Koch, 2005; Jiang and He, 2006; Jiang et al., 2009) can be processed sufficiently for the healthy brain to distinguish neutral from emotional expressions, including fear, disgust, and happiness. Differential brain responses to both invisible and visible facial expressions have been measured, for instance, using functional magnetic resonance imaging (fMRI; e.g., Williams et al., 2004; Jiang and He, 2006) and surface event-related potentials (ERPs; e.g., Jiang et al., 2009; Smith, in press). Thus, it is now widely thought that facial expressions can influence neural activity and behavior independently of awareness, and that they constitute a stimulus class well suited for investigating differences between conscious and non-conscious perception in the human brain.

One fundamental question, which is the focus of the present article, concerns which “low-level” aspects of facial-expression signals modulate brain responses as a function of awareness. Faces are complex stimuli that contain information at various spatial frequencies (SFs). Broadly speaking, low SFs represent the coarse information in an image (e.g., luminance blobs), whereas high SFs represent the fine-grained information (e.g., fine wrinkles in a face). It is well known that the visual system filters any retinal input with multiple quasi-linear band-pass filters, each tuned to a specific range of SFs (see De Valois and De Valois, 1990, for a review). The contribution of different SFs to the perception of facial expressions has been investigated in a number of fMRI (Vuilleumier et al., 2003; Winston et al., 2003b; Morawetz et al., 2011) and surface ERP (Holmes et al., 2005; Pourtois et al., 2005; Schyns et al., 2007, 2009; Vlamings et al., 2009) studies. However, the studies led to mixed findings and were limited in several respects. For instance, the low temporal resolution of fMRI and the low spatial resolution of surface ERPs did not allow for conclusions to be drawn about the precise temporal dynamics of SF processing in specific brain regions. Moreover, the SF filtering methods that were employed (low-pass, high-pass, or band-pass filtering) provided only a crude estimate of SF tuning. Also, the studies were restricted to consciously perceived face stimuli. Therefore, not much is known yet about the “informational correlates” of consciousness in this context – i.e., precisely which SFs are correlated with localized brain signals during the conscious vs. non-conscious perception of emotional expressions.

The aim of the present study was to examine which SFs are correlated with brain signals in specific regions of the emotion-processing network under different awareness conditions. We had the opportunity to record intracranial ERPs from the insula and, to a lesser extent, from the amygdala of patients undergoing monitoring for medically intractable epilepsy. The insula and amygdala have previously been associated with the processing of disgust and fear, respectively (e.g., Adolphs et al., 1994, 1995; Phillips et al., 1997, 1998, 2004; Krolak-Salmon et al., 2003, 2004; but for evidence that the insula also responds to fear, see, e.g., Morris et al., 1998, and for evidence that the amygdala also responds to disgust, see Winston et al., 2003a; Fitzgerald et al., 2006; Van der Gaag et al., 2007). Here, we traced which SFs in disgusted and fearful faces modulate activation in these two interconnected brain structures over time. Our study employed a novel combination of CFS (Tsuchiya and Koch, 2005), intracranial recordings, and the SF Bubbles technique (Willenbockel et al., 2010a). We will elaborate on the three methods in the following paragraphs and briefly review some of their applications in previous studies.

CFS is a powerful method to render visual stimuli invisible (Tsuchiya and Koch, 2005). One of its main strengths is that it allows for suppressing stimuli from awareness for a long duration (i.e., up to several seconds). A second strength of CFS is that the onset of the suppression can be precisely timed. CFS involves presenting a static image to one of the observer’s eyes, while dynamic high-contrast noise (e.g., Mondrian patterns flashed at 10 Hz; Tsuchiya and Koch, 2005) is presented to the other eye. As a result of this dichoptic stimulation, typically only the noise is consciously perceived; the static stimulus is suppressed from the observer’s awareness but nevertheless processed in the brain (e.g., Tsuchiya and Koch, 2005; Jiang and He, 2006; Jiang et al., 2009). Using CFS and fMRI, Jiang and He (2006) found that suppressed fearful compared with scrambled faces elicited significant activation in the fusiform face area, superior temporal sulcus, and the bilateral amygdalae. The amygdalae were also more activated by fearful than by neutral faces, independently of awareness. Using CFS in combination with surface ERPs, Jiang et al. (2009) observed significant amplitude differences to suppressed fearful vs. scrambled faces beginning at 140 ms and to suppressed fearful vs. neutral faces starting at 220 ms after stimulus onset. Overall, combining CFS with fMRI, which has a high spatial resolution, or with surface ERPs, which have a high temporal resolution, has provided important insights into the “where” or “when” of non-conscious facial expression processing – but not both aspects simultaneously. In the present study, we combined CFS with intracranial recordings, which combine some of the advantages of fMRI and surface ERPs.

It has been argued that intracranial recordings currently provide the best combination of high temporal *and* high spatial resolution, plus large anatomical field-of-view and wide frequency bandwidth (Tsuchiya et al., 2008). A number of previous intracranial ERP studies with patients undergoing epilepsy monitoring investigated the temporal dynamics of conscious emotional facial expression processing in the insula and amygdala (Krolak-Salmon et al., 2003, 2004; Pourtois et al., 2010). Krolak-Salmon et al. (2003) found amplitude differences to disgusted vs. neutral, fearful, and happy expressions in the ventral anterior insula. This “disgust effect” started at approximately 300 ms post stimulus onset when observers were engaged in an expression task and approximately 100 ms later when they performed a face-gender task. In a similar study, a “fear effect” was observed in the amygdala, starting at 200 ms in an expression task and later (after 600 ms) in a face-gender task (Krolak-Salmon et al., 2004). Pourtois et al. (2010) observed earlier amplitude differences to fearful vs. neutral faces in the amygdala, starting at 140 ms post stimulus onset. This early effect was not affected by attention but an attentional modulation of emotional responses occurred at longer latencies (after 700 ms). Intracranial ERPs were also used to study amygdala activation to masked emotional words (Naccache et al., 2005). Differences between invisible threatening and neutral words were found after 800 ms post stimulus onset. In the current study, we combined intracranial recordings with CFS to investigate the temporal dynamics of non-conscious emotional expression processing in these brain regions. Furthermore, we went beyond previous studies by examining precisely which SFs in fearful and disgusted faces modulate brain signals over time by combining intracranial recordings with the SF Bubbles technique (Willenbockel et al., 2010a).

The Bubbles method (Gosselin and Schyns, 2001) is a classification image technique that can be used to reveal which stimulus information modulates observers’ behavioral (e.g., Adolphs et al., 2005; Smith et al., 2005) or brain (Schyns et al., 2003, 2007, 2009; Smith et al., 2004, 2007) responses. SF Bubbles (Willenbockel et al., 2010a) is a variant of the technique that can be employed to examine which information in the SF domain correlates with observers’ responses. SF Bubbles involves randomly sampling the energy of visual stimuli at different SFs on a trial-by-trial basis and then performing a multiple linear regression on the information samples and the response measure of interest to precisely reveal the SF tuning curves for a given task. For example, Willenbockel et al. (2010a) used the technique to compare the SF tuning of upright and inverted face identification, and Thurman and Grossman (2011) employed it to investigate SF tuning for discriminating videos of human actions. In the latter study, the results obtained with SF Bubbles were directly compared with those from a more traditional band-pass filtering approach. The results from both methods were consistent but the authors stressed that SF Bubbles offers several advantages. Specifically, SF Bubbles allows for deriving SF tuning curves – spanning the whole SF spectrum – at a much higher resolution and based on a smaller number of trials. A second strength of the method is that randomly sampling multiple SFs simultaneously on a trial-by-trial basis minimizes the risk that participants adapt to a predictable stimulus manipulation (e.g., band-, low-, or high-pass filtering or critical band masking; see Sowden and Schyns, 2006, for evidence of “channel surfing”). Moreover, SF Bubbles is unbiased in that no cutoff frequencies have to be chosen – a parameter that differs considerably between previous experiments using traditional filtering methods (for examples from the emotion-processing literature, see, e.g., Vuilleumier et al., 2003; Vlamings et al., 2009; Morawetz et al., 2011).

The combination of SF Bubbles with intracranial recordings employed in the current study allowed us to map the SF tuning of the insula and amygdala over time. In one condition, we used CFS to render SF filtered disgusted and fearful faces invisible (i.e., dynamic Mondrian patterns were presented to one eye while an “SF bubblized” emotional face was presented to the other eye). In the other condition, the filtered faces were visible (i.e., an “SF bubblized” face was presented to both eyes). Overall, this study represents a unique opportunity to shed light on the neural processing dynamics for ecologically important visual information as a function of awareness.

## Materials and Methods

### Participants

Three patients with medically intractable epilepsy gave their written informed consent and participated in this experiment. The patients were undergoing epilepsy monitoring at the Hôpital Notre-Dame, Montréal, to guide neurosurgical treatment. For this purpose, they had electrodes implanted under a clinical protocol; the electrode locations were chosen solely based on medical considerations. Our study was approved by the CHUM (Centre Hospitalier de l’Université de Montréal) ethics committee and took place at the hospital approximately 6–10 days after the electrode implantation. The participants were naïve to the awareness aspect of the study until the debriefing after the experiment. All of them had normal or corrected-to-normal vision; further participant information is summarized in Table 1.

**Table 1:** **Participant information**.

ID	Gender	Age (years)	Handedness	Seizure focus	Hemisphere recorded from	Number of trials
1	Female	39	Ambidextrous	Frontal operculum Temporal operculum Insula Superior temporal gyrus	Left	1920
2	Male	34	Ambidextrous	Hippocampus	Left	1823
3	Male	35	Right	Inferior frontal gyrus	Right	2007

### Anatomical location of the electrodes of interest

All patients had depth electrodes (Ad-Tech Medical Instrument Corporation, Racine, WI, USA) implanted in the insula, and additional grid, strip, or depth electrodes in other regions. One of the patients had a depth electrode implanted in the amygdala. The implantation schemes are described in detail in a previous article (Surbeck et al., 2011). Patient 1 underwent an open microdissection of the Sylvian fissure (Type I implantation). In the anterior, medial, and posterior insula each, she had a Spencer depth electrode with a diameter of 1.1 mm, which featured four contacts along its length. The contacts were of 2.3 mm in length and spaced 5 mm apart from center to center. Two contacts per electrode ended up in the insular cortex. In the amygdala, she also had a depth electrode with four contacts (1.1 mm diameter, 2.3 mm length, 10 mm spacing). Patient 2 underwent the combined Yale-Grenoble stereotactic implantation (Type II). In the anterior and posterior insula each, he was implanted with a 10-contact Spencer depth electrode (1.1 mm diameter, 2.3 mm length, 10 mm spacing). Patient 3 underwent a Type I implantation with a new hybrid operculo-insular electrode (Ad-Tech Medical Instrument Corporation, WI, USA), among other regular subdural electrodes. The hybrid electrode combined the design of a depth and a subdural strip electrode. The depth component featuring two contacts was implanted into the insular cortex. The length of that segment was 10 mm and the diameter 1.1 mm. The length of each contact was 2.4 mm. Further information can be found in an article by Bouthillier et al. (2012).

**Figure 1:**
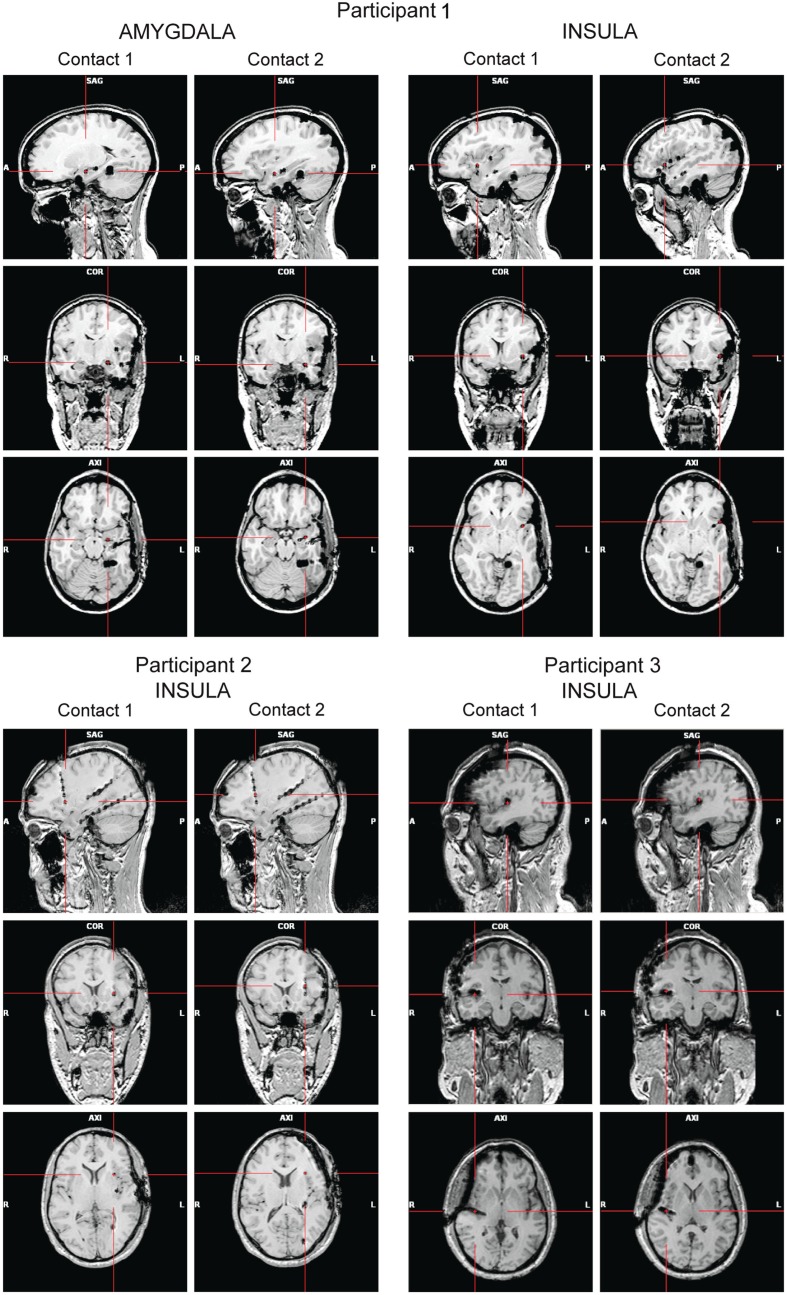
**Panels show the locations of the electrode contacts of interest for each participant based on post-implantation MRIs (A, anterior; P, posterior; L, left; R, right)**.

High-resolution MRIs with 1 mm-thick slices were obtained after the implantation to determine the exact position of the electrodes (Figure 1). A 3D representation of the electrodes with respect to the patient’s brain was generated using Grid View software (Stellate Systems Inc., Montreal, QC, Canada; see also Wang et al., 2005). In the analyses presented here we included two contacts per electrode implanted either in the anterior insula (Participants 1–3) or in the amygdala (Participant 1).

### Electrophysiological recording and stimulus display

Intracranial EEG was recorded at 2 kHz using a Stellate Harmonie system (Stellate Systems, Inc., Montreal, QC, Canada). Either a subdural parietal contact (Participant 1), a subdural temporal contact (Participant 2), or the mastoids (Participant 3) served as a reference. This heterogeneity was not a concern to us because we were interested in the correlations between random SF filters and trial-by-trial voltage variations, which are robust to reference changes. The timing of the stimulus onsets was determined based on the recording of digital trigger signals by the Stellate eAmp using the eAMP Trigger Interface. A dual core 2.19 GHz PC (AMD Athlon 64 X2 4200+) and a 17′′ LCD display (VE700, ViewSonic, CA, USA) were used for presenting the stimuli. The gamma parameter was set to 1, to linearize the relationship between the RGB values and corresponding luminance values. The refresh rate was 60 Hz and the resolution 1024 × 768 pixels. The luminance range in the green channel was diminished to match the red channel, which typically has a lower maximum luminance (min = 0.4 cd/m^2^, max = 33.3 cd/m^2^). All stimuli were shown on a gray background (13.57 cd/m^2^) using the Psychophysics toolbox (Brainard, 1997; Pelli, 1997) for MATLAB (Mathworks, Natick, MA, USA).

### Stimuli

**Figure 2:**
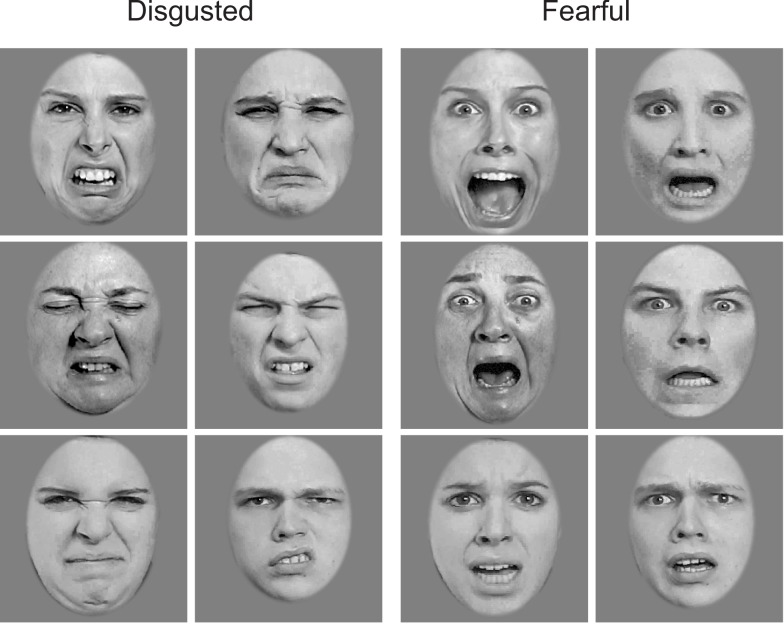
**Base face images with disgusted and fearful expressions used in the experiment**.

Twelve grayscale face photographs (256 × 256 pixels) from the STOIC database (Roy, Roy, Éthier-Majcher, Fortin, Belin, and Gosselin, submitted) served as base stimuli. The photographs depicted three male and three female faces, each with a disgusted and a fearful expression (Figure 2). The faces were cropped to exclude non-facial cues, and they were equated in mean luminance and contrast [root mean square (RMS) contrast of 0.2] using the SHINE toolbox (Willenbockel et al., 2010b).

**Figure 3:**
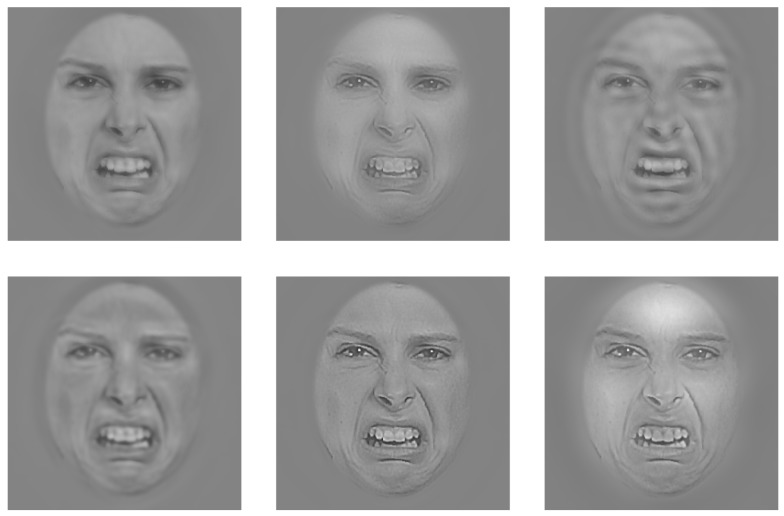
**Example of a face image filtered with the SF Bubbles technique on six hypothetical trials**.

The SFs of the base images (see Figure S1 in Supplementary Material for a plot of the spectral content of the base faces) were randomly sampled trial-by-trial using the SF Bubbles technique (Willenbockel et al., 2010a). In brief, the to-be-filtered base image was padded with a uniform gray background and then subjected to a fast Fourier transform. The amplitude spectrum of the padded image was multiplied element-wise with a filter constructed in the following way: A vector consisting of randomly distributed binary elements (45 ones among 10,195 zeros) was convolved with a Gaussian kernel, referred to as an “SF bubble” (σ = 1.8). This yielded a smoothed sampling vector. The sampling vector was subjected to a logarithmic transformation to take into account the fact that the human visual system is more sensitive to low than to high SFs (e.g., De Valois and De Valois, 1990). To obtain a two-dimensional filter, the log-transformed, smoothed sampling vector was then “rotated” about its origin. After multiplying the two-dimensional filter element-wise with the amplitude spectrum of the base image, the result was back-transformed into the image domain via an inverse fast Fourier transform. The “SF bubblized” image contained a random subset of the base image’s SF content (see Figure 3 for sample stimuli; for an illustration of the filtering procedure, see Figure 1 in Willenbockel et al., 2010a).

The contrast level of the SF sampled stimuli was kept constant across experimental conditions but was adjusted for each participant so he/she reported being able to recognize the facial expressions in the *visible face* condition (see Procedure) but did not detect the faces in the *invisible face* condition. For Participants 1 and 2, this resulted in a mean RMS contrast of 0.019 and for Participant 3 of 0.024. To be able to display stimuli with low contrast, we used Floyd-Steinberg dithering (Floyd and Steinberg, 1976), which enhances the luminance resolution (see also Allard and Faubert, 2008). The face stimuli subtended visual angles of approximately 7.1° horizontally and 12.8° vertically.

**Figure 4:**
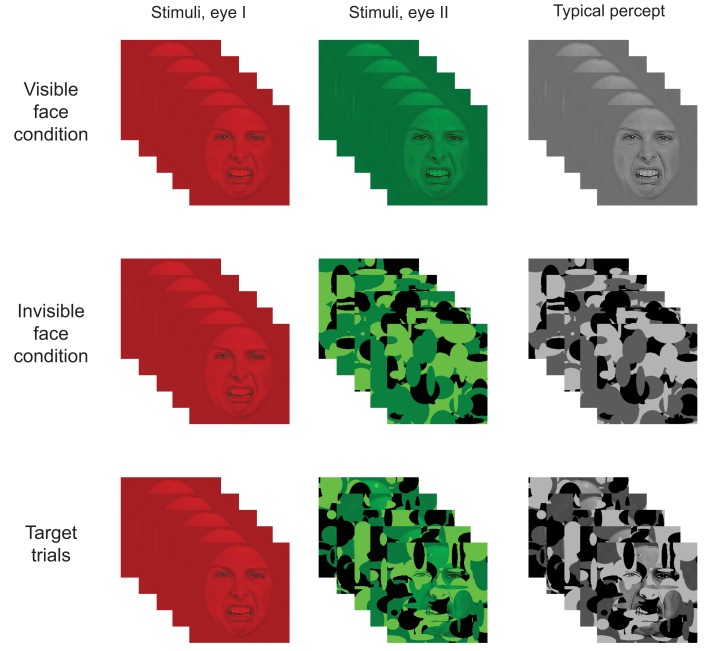
**Illustration of the paradigm**. In the visible face condition, a stationary SF filtered face image was shown to both eyes simultaneously (i.e., in the red and green layers). In the invisible face condition, an SF filtered face was shown to one eye (i.e., in the red layer) while dynamic noise patterns were presented at 10 Hz to the other eye (i.e., in the green layer). Target stimuli consisted of a stationary SF filtered stimulus presented to one eye and a combination of a face and noise patterns to the other eye. As a result, participants typically perceived the face image on visible face trials, only the dynamic noise on invisible face trials, and both face and noise on target trials. (Note that the contrast and brightness of the images was slightly modified in the figure to improve readability.)

The high-contrast noise used for CFS (Tsuchiya and Koch, 2005) consisted of random elliptical Mondrian patterns (Figure 4; see also, e.g., Tsuchiya et al., 2009). The mean RMS contrast of the Mondrians was 0.80 (*SD* = 0.11). The noise fields were of 256 × 256 pixels and subtended horizontal and vertical visual angles of approximately 10.6° and 13.7°, respectively (see Figure S1 in Supplementary Material for a plot of the spectral content of the Mondrians).

Target stimuli consisted of face/Mondrian composites (Figure 4). A composite was constructed by multiplying the pixel values of an SF sampled face image (RMS contrast = 0.04) element-wise with those of a Mondrian noise field and then adjusting the contrast so it matched the Mondrians. For each target trial, five Mondrian/face composites were constructed using the same face image but different Mondrian patterns.

### Procedure

The participants took part in the experiment while sitting comfortably in their dimly lit hospital room. All stimuli appeared at the center of the computer screen and were viewed from a distance of 56 cm through red-green anaglyph glasses. The glasses allowed us to simultaneously present distinct information to each eye of the participant (i.e., one eye with information in red and the other with information in green). Each trial began with a fixation cross presented for 500–900 ms (to both eyes), followed by a blank screen for 500–900 ms. Then, a face stimulus was displayed for 500 ms in one of three conditions: the *invisible face* condition, the *visible face* condition, or the *target* condition (Figure 4).

In the invisible face condition, we employed CFS to suppress the face stimulus from awareness. The static SF sampled face image was presented to one eye (by showing it in the red layer of the RGB image) while the other eye was presented with suppression noise (i.e., Mondrians were presented in the green layer). The Mondrians changed at a rate of 10 Hz (see also Tsuchiya and Koch, 2005). As a result, only the dynamic Mondrians were consciously perceived. In the visible face condition, both eyes were presented with the same SF filtered face by displaying it in both the red and green layers. On target trials, a face was shown to one eye (i.e., in the red layer) while a Mondrian/face composite was shown to the other eye (i.e., in the green layer) at 10 Hz.

The participants were instructed to look at all images carefully and to press the space bar on a regular computer keyboard if they perceived Mondrian patterns and a face together on a given trial. The detection task allowed us to see if participants were paying attention to the stimuli and to evaluate for each CFS trial whether the faces were successfully suppressed. The interstimulus interval was adjusted for each participant to ensure that he/she had enough time for the keypress (see also Jiang and He, 2006).

One experimental session typically consisted of five 105-trial blocks (plus one practice block in the first session), with breaks in between. After each session, the red and green lenses were swapped. 45.7% of the trials were invisible face trials, 45.7% were visible face trials, and 8.6% were target trials. The different trial types were randomly intermixed within each block. We recorded four sessions per participant (with a maximum of two sessions per day, depending on the patient’s willingness for research participation and on clinical constraints). In total, Participant 1 completed 20 blocks, Participant 2, 19 blocks, and Participant 3, 21 blocks.

### Analysis

The intracranial EEG data from all contacts of interest for each participant were segmented from 200 ms before stimulus onset until 1500 ms after stimulus onset and baseline corrected using Brain Vision Analyzer 2.0.1 (Brain Products GmbH, Munich, Germany). The following analyses were carried out with custom MATLAB programs. Target trials and all other trials on which a keypress was made were excluded from the SF analysis. Table 1 provides the exact number of trials included in the analyses for each participant.

To trace which SFs modulate the EEG amplitudes recorded from the insula or amygdala over time, we ran multiple linear regressions on the SF filters from each trial and the transformed EEG amplitudes within time bins of 20 ms (separately for each participant, brain region, condition, and session). EEG amplitudes within a given time bin were transformed as follows: First, we averaged the recorded EEG amplitudes within the time bin and across the two contacts of interest from each electrode. Then we performed a median split across trials: we set the amplitude from a given trial to 1 if it was greater than or equal to the median of all trials or to −1 if it fell below the median. This way, the impact of any abnormal amplitudes (e.g., due to epileptic spikes) was minimized without having to rely on a subjective trial rejection criterion. We then summed the filters from all trials weighted by the transformed amplitudes, which, here, is equivalent to a multiple linear regression. This was done separately for each of the 85 bins between 200 ms before stimulus onset and 1500 ms after stimulus onset.

The vectors of regression coefficients obtained for each time bin were stored in a time segment × SF sampling points matrix and smoothed using Gaussian kernels with a standard deviation of 4.0 time bins and 300 sampling points. The result was transformed into *Z*-scores – henceforth called classification images (CIs). We focus here on the overall CIs for each brain structure (insula and amygdala) and for each awareness condition to maximize the signal-to-noise ratio. Separately for the visible and invisible face conditions, we summed the CIs across sessions and divided the result by the square root of the number of sessions (i.e., √4). We then summed the resulting CIs across emotional expressions and divided by the square root of the number of expressions (i.e., √2). In addition, to compute the insula CIs, we summed the respective CIs across participants and divided by the square root of the number of participants (i.e., √3). Statistical significance was evaluated using the Pixel test from the Stat4Ci toolbox (*p *< 0.05, *S*_r_ = 870400, FWHM* *= 99.91, *Z*_crit_ = ±3.78; Chauvin et al., 2005).

## Results

### Behavioral results

The detection task served two purposes: (a) to ensure that participants stayed alert during the experiment, and (b) to check on each CFS trial whether the face broke through the suppression noise. The percentage of correctly detected targets for the three participants was very high (*M* = 97.07%, *SD* = 1.13%), suggesting that the participants paid attention to the stimuli. The percentage of detected non-targets was small (*M* = 0.30%, *SD* = 0.43%), which confirmed that the faces were successfully suppressed from awareness in the invisible face condition.

### Spatial frequency results

**Figure 5:**
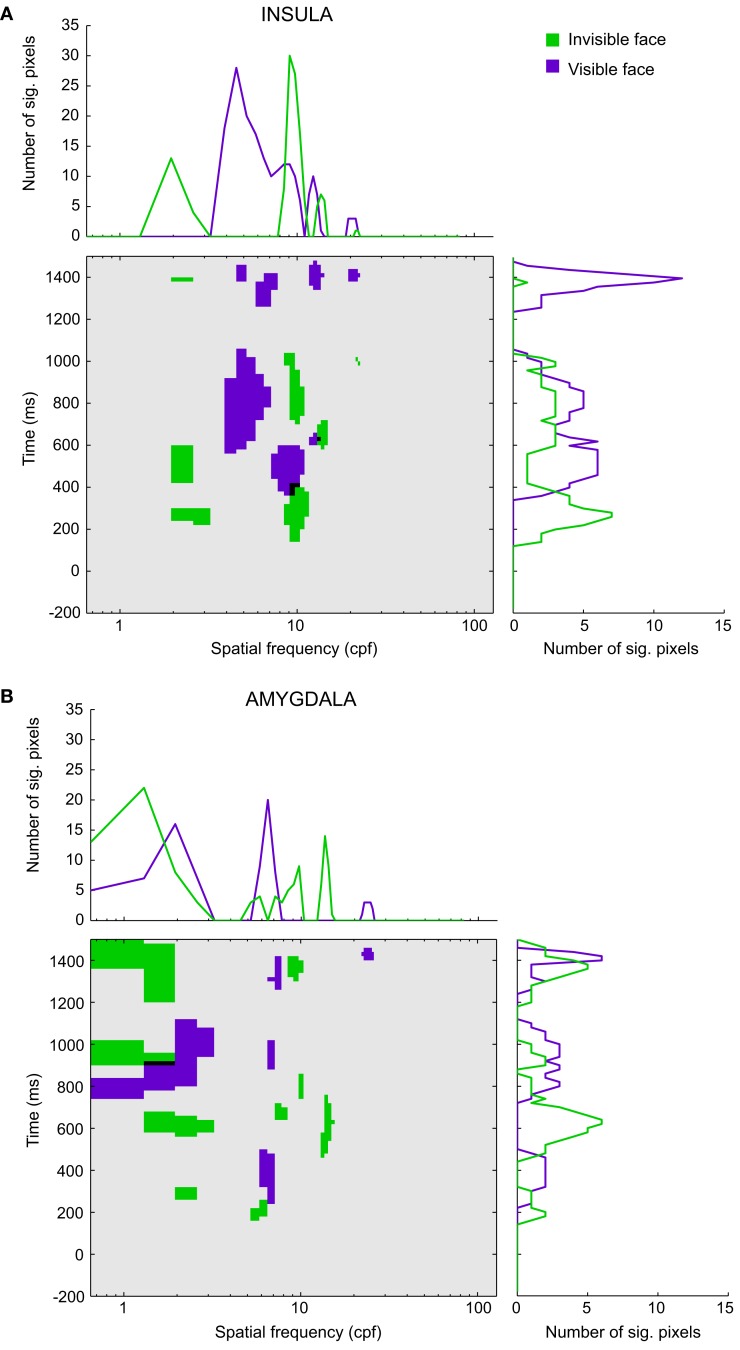
**Classification images for (A) the insula and (B) the amygdala**. The classification images show the significant pixels for the invisible face condition (green) and the visible face condition (purple) for each spatial frequency (in cpf, cycles per face) and time segment between 200 ms before stimulus onset and 1500 ms after stimulus onset. Black regions indicate the overlap between the awareness conditions. The line graphs (summation plots) show the number of significant pixels across time for each spatial frequency (top) or across spatial frequencies for each time segment (right).

Figure 5 depicts the significant pixels (regardless of polarity) for each SF and time bin, up to 1.5 s after stimulus onset for the overall insula and amygdala CIs (see Figure S2 in Supplementary Material for the raw, non-thresholded, CIs). The purple pixels correspond to the visible face condition, the green pixels to the invisible face condition, and the black pixels indicate overlaps between the conditions. We will focus on the SFs that reached significance during stimulus presentation (0–500 ms). Note, however, that for both regions and visibility conditions, we found multiple other low-, mid-, and high-SF clusters to be significant after the offset of the stimulus.

Figure 5A shows the results for the insula (Participants 1–3). In the visible face condition, SFs around 8.75 cycles per face width (cpf) reached significance at approximately 340 ms after stimulus onset. In the invisible face condition, SFs around 9.40 cpf became significant at approximately 140 ms, followed by very low SFs around 2.27 cpf. The latter attained significance at approximately 200 ms and again at 420 ms. The significant pixels of the two visibility conditions overlap for SFs around 9.04 cpf between 340 and 400 ms.

Figure 5B displays the results for the amygdala (Participant 1). In the visible face condition, SFs around 6.48 cpf attained significance at approximately 240 ms. In the invisible face condition, SFs around 5.51 cpf became significant at about 140 ms. Then, at approximately 260 ms, very low SFs (1.95 cpf) reached significance.

The line graphs (summation plots) on top of the CIs depict the number of significant pixels for each SF, collapsed across time. For both the insula and amygdala, they show quite clearly that processing in the invisible face condition relied on low SFs more than processing in the visible face condition. Likewise, the graphs on the right of the CIs show the number of significant pixels for each time bin, collapsed across SFs. For both regions, they indicate that significant correlations between SFs and brain signals occurred earlier for the invisible than for the visible face condition.

## Discussion

The aim of the present study was to shed light on the informational correlates of consciousness in the context of emotional facial expression perception. Specifically, we examined which SFs in consciously and non-consciously perceived stimuli are correlated with brain signals in two key structures of the emotion-processing network – the insula and the amygdala. We employed a novel combination of three techniques: intracranial recordings in awake human participants, SF Bubbles (Willenbockel et al., 2010a), and CFS (Tsuchiya and Koch, 2005). To our knowledge, this is the first study that mapped the time course of SF tuning for specific regions of the emotion-processing network and as a function of awareness. In the following, we will put our findings into context by focusing, in turn, on (a) emotional expression perception and awareness, (b) awareness and SF processing (during face perception in general), and (c) SF processing and emotional expression perception. We will then briefly discuss our findings in light of theories on the neural pathways involved in emotion perception.

Disgusted and fearful faces were used as stimuli because previous work has shown that the insula and amygdala are implicated in the processing of these facial expressions. In particular, numerous studies led to the conclusion that the anterior insula is important for the processing of disgust (e.g., Phillips et al., 1997, 1998; Krolak-Salmon et al., 2003), and the amygdala for the processing of fear (e.g., Adolphs et al., 1994, 1995; Morris et al., 1996; Phillips et al., 1998; Krolak-Salmon et al., 2004); taken together, other studies indicated that these brain regions respond to both disgusted and fearful faces (e.g., Morris et al., 1998; Winston et al., 2003a; Fitzgerald et al., 2006; Van der Gaag et al., 2007; see also Anderson et al., 2003). Our results replicate these findings. However, the emotion-specificity of the responses in these regions goes beyond the scope of this article.

Previous work has also shown that both disgusted and fearful expressions can be perceived independently of awareness (e.g., Smith, in press). Using various methods to render stimuli invisible, neuroimaging studies demonstrated that the amygdala is involved in the non-conscious processing of emotional faces (e.g., Whalen et al., 1998; Morris et al., 1999; Pasley et al., 2004; Williams et al., 2004, 2006; Jiang and He, 2006; but see Phillips et al., 2004). Scarce studies found support for an involvement of the insula in the non-conscious processing of emotional stimuli (e.g., Sabatini et al., 2009; but see, Anderson et al., 2003, and Phillips et al., 2004, for results that speak against automatic facial expression processing in the insula). The present results indicate that both structures play a role in perceiving emotional expressions, consciously and non-consciously.

In our visible face condition, the first significant correlations between stimulus information and brain signals occurred at approximately 340 and 240 ms after stimulus onset in the insula and amygdala, respectively. In our invisible face condition, they were present as early as 140 ms in both regions. Moreover, in both visibility conditions, we found significant correlations at long latencies, up to 1500 ms after stimulus onset. These temporal dynamics appear largely consistent with the results from previous intracranial ERP studies on conscious emotional facial expression perception, although a direct comparison is difficult due to important methodological differences. In line with our finding that the response of the insula occurred later than that of the amygdala, previous results revealed emotional effects as early as 300 ms post stimulus onset in the insula (Krolak-Salmon et al., 2003), and as early as 140 (Pourtois et al., 2010) or 200 ms (Krolak-Salmon et al., 2004) in the amygdala.

Furthermore, long-latency effects were present in previous intracranial ERP data as well. For instance, Krolak-Salmon et al. (2004) observed differential responses to fear vs. neutral or happy faces until 1100 ms after stimulus onset in the amygdala of one patient. Pourtois et al. (2010) found late emotional effects in the amygdala that were modulated by attention, starting at approximately 700 ms after stimulus onset and lasting more than 300 ms. Finally, such late effects were seen in response to invisible emotional words in the amygdala (after 800 ms after stimulus onset; Naccache et al., 2005), suggesting that considerable time is needed for extracting emotional meaning. Naccache et al. (2005) speculated that top-down influences might amplify non-conscious amygdala activation in this context, without making information accessible to conscious report. Possibly, the late significant correlations with low-level information that we found also reflect feedback or top-down influences that amplify certain aspects of the stimuli later on.

Our CIs show complex patterns of SF tuning over time, for both the insula and the amygdala. A comparison of the CIs between awareness conditions revealed that invisible face processing relied on very low SFs (<3 cpf) more than visible face processing, especially within the first 600 ms after stimulus onset. The idea that SF processing and awareness interact during face perception has come up repeatedly in the literature but has, as far as we know, only been investigated in one published study (De Gardelle and Kouider, 2010). The authors employed a masked priming paradigm with hybrid prime stimuli – composed of the low SFs of one face and the high SFs of another face – and a fame judgment task. Using behavioral measures, they discovered that both low SFs (<12 cpf) and high SFs (>12 cpf) could be processed without awareness. The influence of high SFs correlated with prime visibility (i.e., prime duration), whereas the influence of low SFs did not. De Gardelle and Kouider’s results are consistent with ours inasmuch as we also found a broad range of SFs to be processed non-consciously. The qualitative differences that we observed between our awareness conditions, however, were not seen in their data. This discrepancy could be due to several methodological differences between the studies.

Whereas not much work has been done on SF processing and awareness, several studies have looked at SF processing during the conscious perception of fearful faces. The majority of studies imply that low SFs are particularly important for the perception of fear (Vuilleumier et al., 2003; Winston et al., 2003b; Pourtois et al., 2005; Vlamings et al., 2009; but see Holmes et al., 2005; Morawetz et al., 2011). For instance, in an fMRI study, Vuilleumier et al. (2003) observed larger amygdala responses to fearful than to neutral faces when stimuli were unfiltered or low-pass filtered (<6 cpf), but not when they were high-pass filtered (>24 cpf). In a recent surface ERP study (Vlamings et al., 2009), it was found that fearful relative to neutral faces elicited a larger P1 component (i.e., a positive deflection around 100 ms post stimulus onset) and a larger N170 (i.e., a negative deflection around 170 ms), also only for low-pass (≤12 cpf), not for high-pass (≥36 cpf), filtered faces. These findings are in line with our amygdala CI: many pixels reached significance for SFs under 6 cpf but very few attained significance for SFs above 24 cpf [see the summation plot in Figure 5B (top)]. However, as discussed above, we did not find any significant SFs for latencies below 200 ms in our visible face condition, suggesting that the early emotional effects observed using surface ERPs (Vlamings et al., 2009; see also Pourtois et al., 2005) are probably not driven by the amygdala or insula.

The SF tuning patterns we found raise the question about the underlying neural mechanisms of SF processing as a function of awareness. Specifically, through which pathways does SF information arrive at the insula and amygdala? Currently two theories are discussed in the emotion-processing literature, namely the subcortical pathway hypothesis (for recent reviews see Tamietto and De Gelder, 2010; De Gelder et al., 2011) and the multiple waves model (Pessoa and Adolphs, 2010, 2011). According to the former, low-SF information from emotional stimuli is conveyed quickly and automatically via a subcortical route through the superior colliculus and the pulvinar nucleus of the thalamus to the amygdala, whereas high SFs are processed more slowly along a cortical route. The multiple waves model, in contrast, suggests that emotional information is processed in parallel by multiple cortical pathways, without reliance on a direct subcortical route to the amygdala. Our study was not designed to test these theories; however, our results appear to be consistent with the multiple waves model, while they challenge the subcortical pathway hypothesis in at least two ways. The first hurdle for the subcortical pathway hypothesis is that the early low-SF clusters revealed to be significant in the invisible face condition are *not* present in the visible face condition. The second hurdle is that the latencies we found in both awareness conditions (140 ms in the invisible face condition, and 340 ms or 240 ms in the visible face condition for the insula and amygdala, respectively) do not appear faster than cortical visual processing (see Pessoa and Adolphs, 2010, for a review). More work will be needed to test these two theories.

One limitation of the current study is that since we recorded brain signals from patients with epilepsy, we cannot be entirely sure that our data are representative of the healthy population. For Participant 1, epileptic spikes were found in the insula; we therefore recomputed our CIs without her data for the insular contacts. However, we did not find any changes in the main results (see Figure S3 in Supplementary Material). The structures we recorded from in all participants were structurally normal-appearing on high-resolution MRI. Thus, we think it is reasonable to assume that the results we report here can be generalized. Intracranial recordings from volunteers with epilepsy have previously been used in several studies (e.g., Oya et al., 2002; Krolak-Salmon et al., 2003, 2004; Naccache et al., 2005; Tsuchiya et al., 2008; Pourtois et al., 2010) because they bear a number of advantages – specifically, a millisecond temporal resolution combined with a high spatial resolution – and are thus considered to provide an important window into the workings of the human brain.

A second drawback is that in creating the two awareness conditions, we introduced differences in physical stimulation. In the visible face condition, a static face was presented to both eyes, whereas in the invisible face condition, dynamic high-contrast noise replaced the face presented to one eye. This has the disadvantage that we do not know to what extent and how the flashing of the noise patterns influenced our SF results (see Yang and Blake, 2012). We chose suppression noise that was used in several previous studies and found to be very effective (i.e., high-contrast Mondrian patterns; e.g., Tsuchiya and Koch, 2005; Jiang and He, 2006; Jiang et al., 2009). The spectral energy of our Mondrians was highly correlated with that of our base faces (see Figure S1 in Supplementary Material; the correlation between the average across faces and the average across noise patterns was *r* = 0.95). Our Mondrians consisted of elliptical elements (see also Tsuchiya et al., 2009; Adams et al., 2010) and thus contained energy at all orientations. It is not yet known what the optimal suppression noise would be, and basically all methods used to render visual stimuli invisible for normal-sighted observers introduce differences in stimulation. Therefore, this problem is difficult to overcome (see, e.g., the review by Tamietto and De Gelder, 2010). We used CFS because it results in longer suppression than other techniques, such as backward masking or binocular rivalry. Also, it has the advantage that the suppression can be precisely timed.

Investigating the informational correlates of consciousness from several angles – i.e., with different awareness-manipulating techniques and paradigms (e.g., Faivre et al., 2012; for a review see Kim and Blake, 2005) – might currently be the best approach to overcome the limitations of the present study. For example, one could combine the SF Bubbles technique with masked priming to examine which SFs of visible vs. invisible primes influence observers’ responses to a visible target. It might also be a good idea to use SF Bubbles together with a crowding paradigm, which has recently been emphasized as a more ecologically valid approach than masking or CFS (Faivre et al., 2012). Furthermore, it might be insightful to combine SF Bubbles with an attentional blink (e.g., Raymond et al., 1992) paradigm, where physical stimulation remains constant but stimuli can be rendered invisible by diverting the observers’ attention. This could represent a promising avenue for contrasting SF tuning between conscious and *preconscious* processing (see Dehaene et al., 2006). The present study is meant as a first step toward gathering converging evidence about the informational correlates of consciousness.

## Conclusion

Using state-of-the art techniques, we mapped the SF tuning of the insula and amygdala as a function of awareness. Our results are consistent with the idea that a wide range of SFs plays a role in the conscious and non-conscious perception of emotional facial expressions, with SFs between 6 and 10 cpf appearing particularly important early on (for faces subtending approximately 7°). That being said, qualitative differences in SF tuning were observed between our awareness conditions – particularly in the early processing of very low SFs – that are consistent with the idea that different neural pathways are employed for conveying visual information to the amygdala and insula under different degrees of awareness. The present study paves the way for future work that investigates the temporal dynamics of SF processing in specific structures of the emotion-processing network and for elucidating the informational correlates of consciousness in general.

## Conflict of Interest Statement

The authors declare that the research was conducted in the absence of any commercial or financial relationships that could be construed as a potential conflict of interest.

## Supplementary Material

The Supplementary Material for this article can be found online at http://www.frontiersin.org/Consciousness_Research/10.3389/fpsyg.2012.00237/abstract

Supplementary Figure S1 **Rotational average of the power spectra of the 12 base faces and the suppression noise (540 Mondrian patterns)**.

Supplementary Figure S2 **Raw classification images for the two regions (top: insula; bottom: amygdala) and awareness conditions (left: visible face; right: invisible face)**.

Supplementary Figure S3 **Insula classification images [(A), thresholded; (B), raw] computed from the data of Participants 2 and 3 only**.
