# Supplementary material for: Spatial Frequency Tuning during the Conscious and Non-Conscious Perception of Emotional Facial Expressions – An Intracranial ERP Study
Source: Front Psychol. 2012 Jul 19;3:237. doi: 10.3389/fpsyg.2012.00237 (PMC3458489; doi:10.3389/fpsyg.2012.00237)
Supplement: Supplementary Figure S1 — Rotational average of the power spectra of the 12 base faces and the suppression noise (540 Mondrian patterns). [file Presentation 1.PDF]

Supplementary Materials
for

Spatial frequency tuning during the conscious and non-conscious perception of emotional facial expressions—an intracranial ERP study

Verena Willenbockel[1], Franco Lepore[1], Dang Khoa Nguyen[2], Alain Bouthillier[2], Frédéric Gosselin[1*]

[1]Centre de Recherche en Neuropsychologie et Cognition, Département de Psychologie, Université de Montréal, Montréal, QC, Canada.
[2]Centre Hospitalier de l'Université de Montréal, Hôpital Notre-Dame, Montréal, QC, Canada.

Supplementary Figures 1–3

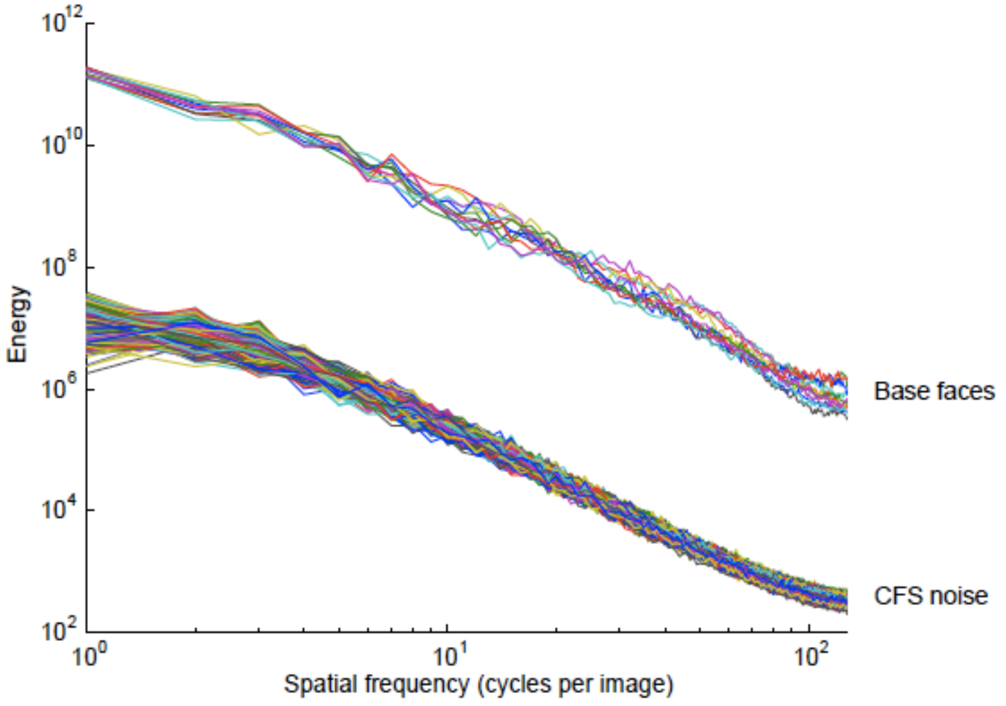

Supplementary Figure 1

Rotational average of the power spectra of the 12 base faces and the suppression noise (540 Mondrian patterns).

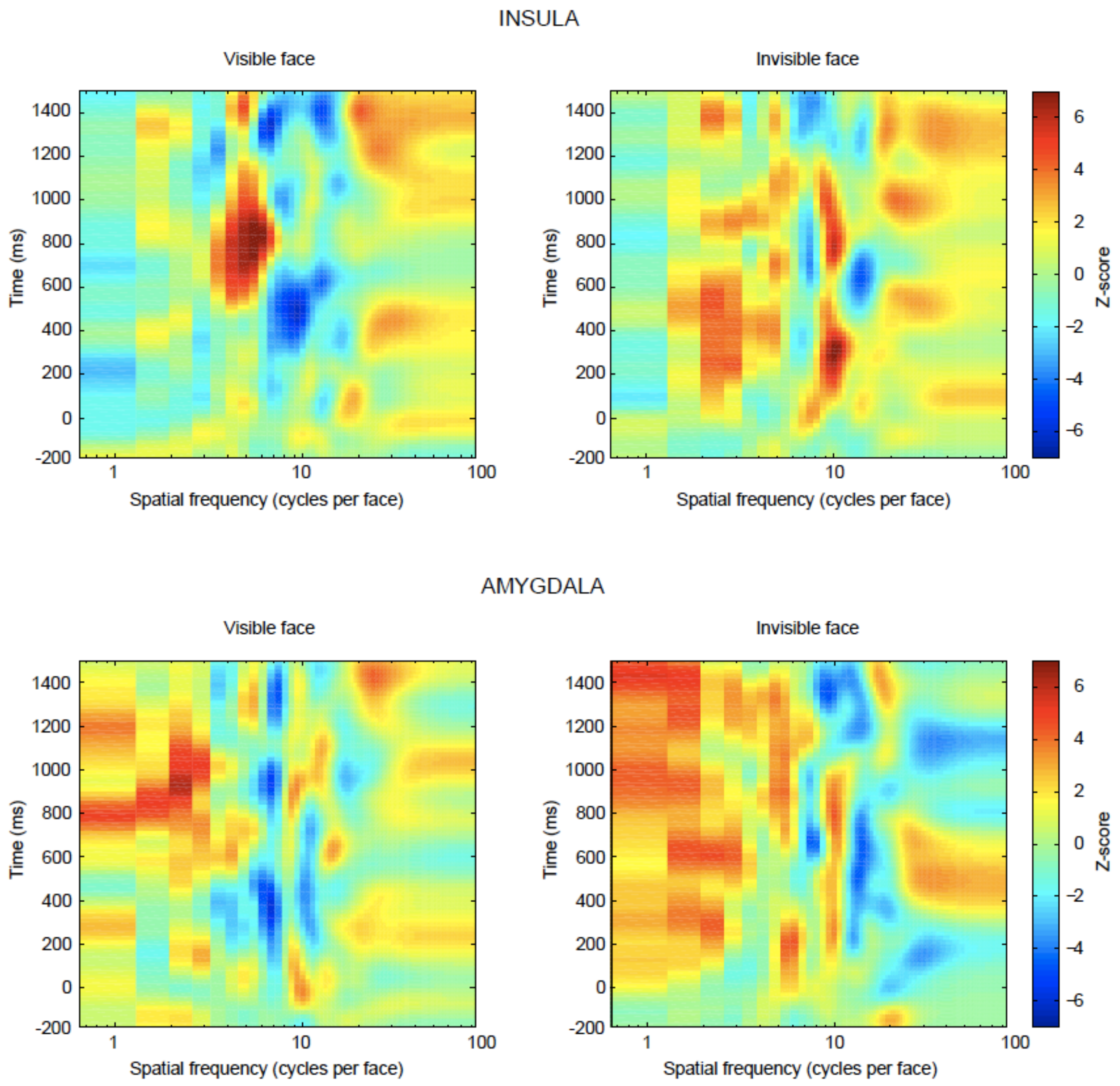

Supplementary Figure 2

Raw classification images for the two regions (top: insula; bottom: amygdala) and awareness conditions (left: visible face; right: invisible face).

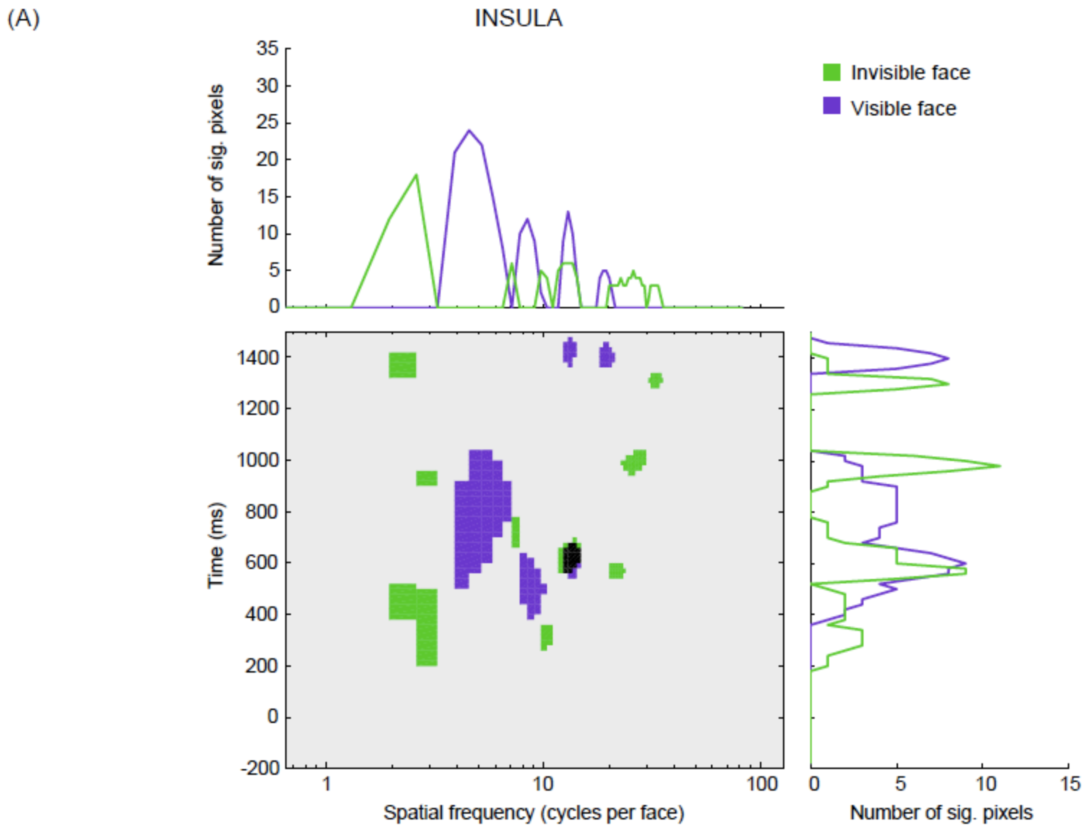

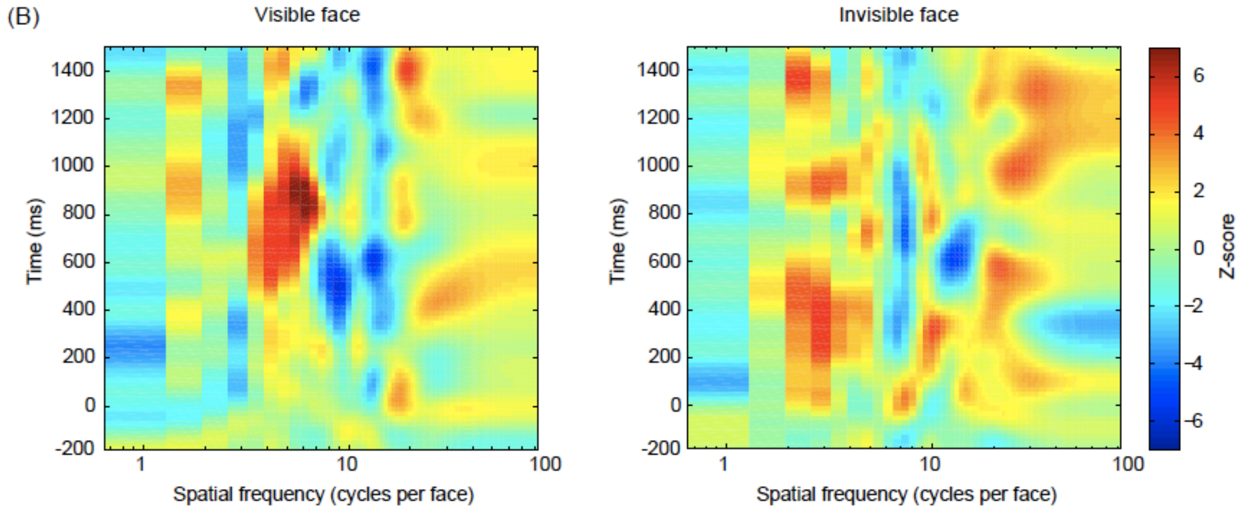

Supplementary Figure 3

Insula classification images (A: thresholded; B: raw) computed from the data of Participants 2 and 3 only.
